# Equine Granulocytic Anaplasmosis: A Systematic Review and Meta-Analysis on Clinico-Pathological Findings, Diagnosis, and Therapeutic Management

**DOI:** 10.3390/vetsci11060269

**Published:** 2024-06-13

**Authors:** Andreea Monica Bogdan, Ioan Liviu Mitrea, Mariana Ionita

**Affiliations:** Department of Parasitology and Parasitic Diseases & Animal Biology, Faculty of Veterinary Medicine, University of Agronomic Sciences and Veterinary Medicine of Bucharest, 011464 Bucharest, Romania; andreeabogdan01@yahoo.com (A.M.B.); ionitamary@yahoo.com (M.I.)

**Keywords:** *Anaplasma phagocytophilum*, equine granulocytic anaplasmosis, clinico-pathology, diagnosis and treatment

## Abstract

**Simple Summary:**

Equine granulocytic anaplasmosis (EGA) is a tick-borne disease affecting horses worldwide, caused by *Anaplasma phagocytophilum.* The disease ranges from non-specific clinical signs to fatal outcomes. A large number of horses has been tested for serological and molecular surveys concerning EGA, but the number of clinical EGA reports is smaller and mostly as single or case series reports. Therefore, the aim of this paper was to analyze reported cases regarding clinico-pathology, diagnosis, and therapeutic management in equids that developed clinical signs of EGA and were confirmed to be infected with *A. phagocytophilum*. The variety of clinical and pathological findings and the challenging therapeutic approaches registered strongly suggest that EGA should be included in the differential diagnosis when fever occurs. In addition, the findings emphasize the importance of monitoring EGA to initiate proper medication in order to avoid complications.

**Abstract:**

Equine granulocytic anaplasmosis (EGA) is a tick-borne disease affecting horses worldwide, caused by *Anaplasma phagocytophilum*. The disease ranges from non-specific clinical signs to fatal outcomes. This paper aimed to analyze EGA cases reported in peer-reviewed journals, particularly on clinico-pathological findings, diagnosis, and therapeutic management. Overall, 189 clinical cases from 31 publications were included in the study. Extensive symptomatology for the EGA cases was reported, of which mostly was fever (90.30%), followed by limb edema (48.51%), anorexia (41.79%), depression (32.84%), icterus (22.39%), ataxia (17.91), tachycardia (16.42%), and lethargy (15.67%). Laboratory tests revealed thrombocytopenia (90.32%), anemia (75%), decreased hematocrit (70.59%), leukopenia (55.88%), lymphopenia (58.14%), and neutropenia (41.67%) as the most common hematological abnormalities. For a subset of tested animals, hyperbilirubinemia (20/29), hyperfibrinogenemia (13/15), and hyponatremia (10/10) were also reported. The diagnosis was established by microscopic identification of morulae (in 153 cases), and/or PCR (120 cases), isolation (1 case), or serology (56 cases). For treatment, oxytetracycline was used in the majority (52.24%) of EGA cases, but recovery without antibiotherapy (10.34%) was also noted. In conclusion, the variety of clinical and pathological findings and the challenging therapeutic approaches reported suggest that EGA should be included in the differential diagnosis when fever occurs.

## 1. Introduction

Equine granulocytic anaplasmosis (EGA) is a tick-borne infection caused by *Anaplasma phagocytophilum*, an obligate intracellular Gram-negative bacterium (order Rickettsiales; family Anaplasmataceae) [1,2,3]. The causative species was previously known as *Ehrlichia phagocytophila*, *Ehrlichia equi*, and human granulocytic ehrlichiosis agent, which have been classified into a single species based on the similarities observed between them [4]. Supporting this theory, a human pathogenic strain of *A. phagocytophilum* has been identified in the blood of a horse [5].

The infections caused by *A. phagocytophilum*, namely human granulocytic anaplasmosis (HGA) and animal granulocytic anaplasmosis (equine, canine, and feline granulocytic anaplasmosis, or tick-borne fever in ruminants), are among the most widespread tick-borne infections in animals in Europe. Moreover, *A. phagocytophilum* is also regarded as an emerging human pathogen with growing importance in the Northern Hemisphere [4,6].

EGA was reported for the first time in 1969 in California, USA. In North America, it is transmitted by *Ixodes pacificus* (in California and other western states) and *Ixodes scapularis* (in the eastern and northern Midwest of the United States) ticks [7,8,9].

In Europe, the vector for granulocytic anaplasmosis is *Ixodes ricinus*, the sheep tick [9]. However, *A. phagocytophilum* was also detected in other tick species: *Ixodes crenulatus, Hyalomma* spp. hemolymph and *Rhipicephalus* spp. hemolymph [10,11]. The pathogen is transmitted transstadially; transovarial transmission is rare, and it is considered to be inefficient [12,13]. Besides transmission via tick bite, intrauterine transmission of *A. phagocytophilum* has been reported in bovine and sheep [14,15].

The reservoir hosts for this bacterium are considered to be small mammals, wild boar, cervids, and sheep, while humans, dogs, and horses are considered accidental hosts [9,16,17]. Recently, it has been noted that *Marmota himalayana* (Himalayan marmot) could serve as a reservoir for *A. phagocytophilum* [10].

*A. phagocytophilum* has been reported worldwide in a variety of hosts. In Romania, the pathogen has been detected in ticks, red foxes, small mammals, and wildlife hosts (mammal hosts, birds, and hedgehogs) [18,19,20,21,22,23]. Antibodies against *Anaplasma* spp. have been recently reported in cattle [24]. Also, a recent serological survey conducted by the authors reported, for the first time in Romania, anti-*A. phagocytophilum* antibodies in horses [25].

A large number of horses has been tested using serological and molecular surveys concerning EGA, but the number of clinical EGA reports is smaller and mostly as single or case series reports. Therefore, the aim of the present systematic review and meta-analysis was to analyze clinical cases reported in peer-reviewed journals regarding clinico-pathology, diagnosis, and therapeutic management in horses that developed clinical signs of EGA and were confirmed infected with *A. phagocytophilum*.

## 2. Materials and Methods

### 2.1. Search Methodology, Inclusion and Exclusion Criteria

Publications selected for this systematic review and meta-analysis were found in the PubMed and Google Scholar databases. PRISMA guidelines were used to select eligible articles. For this, two authors (A.M.B. and M.I.) searched independently, using keywords and expressions like *Anaplasma phagocytophilum*, anaplasmosis, equine anaplasmosis, equine granulocytic anaplasmosis, *Anaplasma phagocytophilum* in horses, anaplasmosis in horses, *Ehrlichia equi*, ehrlichiosis, equine ehrlichiosis, and ehrlichiosis in horses. ‘My ncbi filters’ were used to select the results by year and the type of articles needed (i.e., case reports). Once a title has been selected, the abstract was checked to select articles eligible for inclusion. Available full texts of the selected publications were reviewed, including their references, in order to include additional articles that might have been selected in the initial literature search.

The following inclusion criteria were established:

- Peer-reviewed articles containing original research;
- Written in English;
- Publications from 1969 (first case of EGA) until December 2022;
- Available abstract and/or full-text articles;
- Clinical cases of EGA with a description of symptomatology;
- Clinical cases of EGA with confirmatory diagnosis by microscopic and/or molecular methods (polymerase chain reaction).

Publications that did not fulfill the above criteria were excluded.

### 2.2. Information Extracted

In a Microsoft^®^ Excel^®^ 2019 MSO (16.0.15831.20098) spreadsheet, the following information was extracted from the selected articles: country where the cases were reported, continent, number of clinical cases, description, anamnesis, symptomatology, pathological findings, diagnostic methods, therapy, outcome, and reference.

## 3. Results

**Figure 1:**
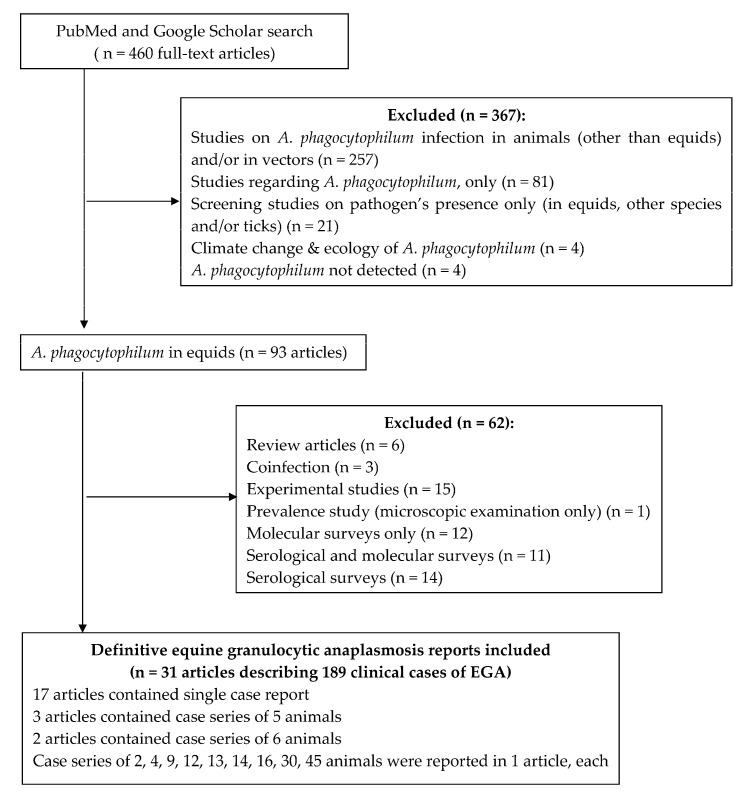
Prisma flow chart for selecting eligible articles for clinical equine granulocytic anaplasmosis (EGA).

The bibliographic research using the above-mentioned criteria resulted in a total of 460 accessed full-text articles; of them, 257 described the pathogen’s occurrence in animals (other than equids) and/or in vectors (ticks, fleas, and lice), 81 were studies regarding *A. phagocytophilum*, only 21 reported *A. phagocytophilum* in equids, other species and/or ticks, 4 emphasized the importance of climate change and ecology in its appearance, 4 were publications in which the bacterium had not been detected in equids or other species, and 93 described its occurrence in equids only. Of the last 93 publications, 31 reported clinical signs of EGA in 189 equids. The PRISMA flow diagram is presented in Figure 1. Therefore, in the present study, a total number of 189 clinical EGA cases described in 31 publications were used to synthesize and analyze findings on EGA with regard to (i) clinical signs and laboratory abnormalities; (ii) diagnosis; (iii) therapy and the outcome. 

### 3.1. Data about the Period and Originating Area of Reported EGA Clinical Cases

**Figure 2:**
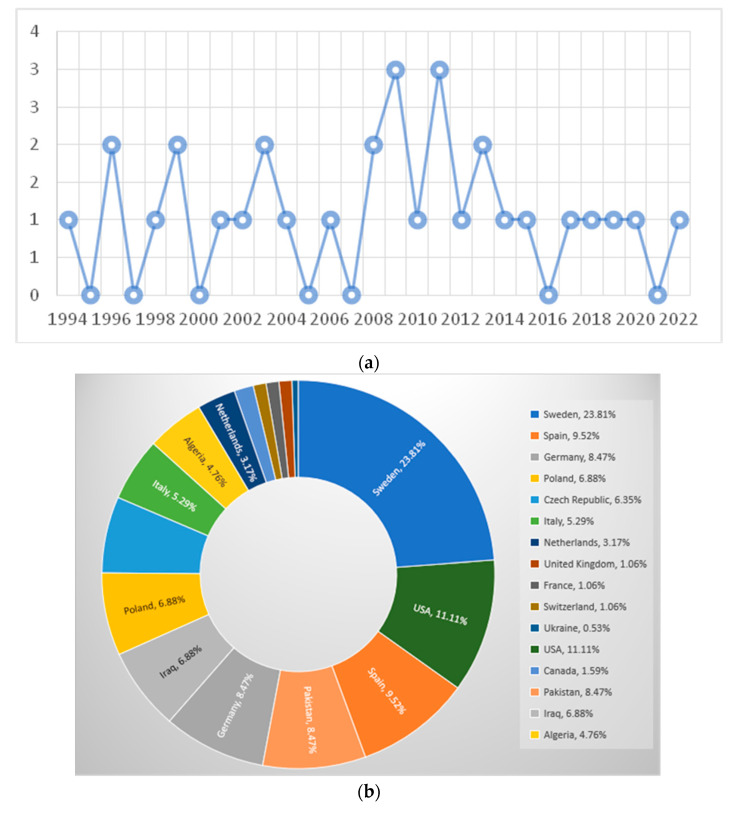
(**a**) Number of published articles per year reporting equine granulocytic anaplasmosis cases. (**b**) Map with the countries where the equine granulocytic anaplasmosis (EGA) reported cases (n = 189) originated from.

Overall, EGA clinical cases were reported from 1994 to 2022 (Figure 2a) on four continents: Europe (127/189; 67.20%), Asia (29/189; 15.34%), America (24/189; 12.70%), and Africa (9/189; 4.76%) (Table 1, Figure 2b).

Of the EGA clinical cases, most were reported from Sweden (n = 45; 45/189; 23.81%), the USA (21/189; 11.11%), Spain (n = 18; 18/189; 9.52%), Germany, Pakistan (n = 16; 16/189; 8.47%, each), Poland, Iraq (n = 13; 13/189; 6.88%, each), Czech Republic (n = 12; 12/189; 6.35%), Italy (n = 10; 10/189; 5.29%), Algeria (n = 9; 9/189; 4.76%), and The Netherlands (n = 6; 6/189; 3.17%). A lower number of cases were reported from Canada (n = 3; 3/189; 1.59%), the United Kingdom, France, Switzerland (n = 2; 2/189; 1.06%, each), and Ukraine (n = 1; 1/189; 0.53%). The complete list of countries where EGA clinical cases were reported is presented in Figure 2b.

### 3.2. General Data about the Animals Diagnosed with EGA

Of the total 189 equids diagnosed with EGA, 179 were horses (94.71%), 6 were donkeys (3.17%), and 4 were mules (2.12%). The animal age was provided for 94 out of 189 cases (49.74%) and varied from 4 months to 30 years old, while sex was reported for 83 (83/189; 43.92%), with 66.27% (55/83) males and 33.73% (28/83) females. More details are provided in Table 1.

### 3.3. Clinical and Laboratory Findings

Equids diagnosed with EGA developed extensive symptomatology. Of the 189 cases, 134 (134/189; 70.90%) had well-defined clinical findings described. One article reported, without any other details on clinical signs for EGA in five horses [33], and two other articles described clinical signs in general for all 16 and 13 cases, respectively, without specifying the number of animals having a certain symptom [11,52]. For the 134 clinical cases, the most commonly registered clinical signs were fever (121/134; 90.30%), followed by limb edema (65/134; 48.51%) and anorexia (56/134; 41.79%) (Table 2). Depression (44/134; 32.84%), icterus (30/134; 22.39%), ataxia (24/134; 17.91%), tachycardia (22/134; 16.42%), and lethargy (21/134; 15.67%) were also frequently reported. Less commonly reported clinical signs were reluctance to move (6/134; 4.48%), abnormal movements (6/134; 4.48%), decreased body weight (5/134; 3.73%), and debilitation (5/134; 3.73%). EGA was also reported in a case with rhabdomyolysis and in two cases with cavitary effusion, which led to cardio-respiratory compromise [46,50]. Despite the fact that EGA is considered an acute illness, one horse developed a fatal chronic cerebral anaplasmosis with clinical signs like loss of consciousness, strabismus, nystagmus, tachypnea, intensified gait disorders, and recumbency [41].

Pathological findings were reported in 124 out of 189 cases (65.61%). In two reports (with 12 and 13 cases, respectively), laboratory results are described in general, without specifying the number of animals having a certain test result [11,52]. For the remaining 99 cases (99/189; 52.38%), hematological tests (for 98/99; 98.99%), differential blood counts (for 45/99; 45.45%), and biochemical assays (for 40/99; 40.40%) were performed. However, the number of determined parameters varied from study to study. Subsequently, hematological tests revealed mostly thrombocytopenia, which was reported in 84 cases (84/93; 90.32%). Other abnormalities frequently reported were anemia (75%; 30/40), decreased PCV/ hematocrit (70.59%; 24/34), and leukopenia (55.88%; 19/34). Differential blood counts most commonly revealed lymphopenia (58.14%; 25/43) and neutropenia (41.67%; 10/24). From the biochemical assays, hyperbilirubinemia (20/29; 68.97%) was reported the most, followed by hyperfibrinogenemia (13/15; 86.67%) and hyponatremia (10/10; 100%). In the case associated with rhabdomyolysis, urinalysis revealed myoglobinuria and proteinuria [46].

Details and a complete list of clinical signs and laboratory/pathological findings are provided in Table 2.

### 3.4. Diagnostic Assays

In the eligible analyzed studies, EGA was diagnosed using one or more of the four following diagnostic methods: microscopy, PCR, isolation, and/or serology. The summarized methods used for diagnosis of the 189 clinical cases of EGA are presented in Table 3.

The microscopic examination allows the detection of characteristic inclusions, so-called morulae, in granulocytes of the infected animals on different types of smears (blood, buffy-coat, pericardial fluid, pleural effusion, cytocentrifuge preparation of the abdominal fluid, mucus sample of the endotracheal tube). This test was carried out for 173 cases (173/189; 91.53%); 153 samples (153/173; 88.44%) were positive, and 20 samples (20/173; 11.56%) were negative.

Of the positive samples, morulae were detected mostly on blood smear (115/153; 75.16%) and buffy coat (32/153; 20.92%). In four cases, characteristic inclusions were observed in both types of smears: buffy coat + blood smears, in two cases (2/153; 1.31%), cytocentrifuge preparation of the abdominal fluid + blood smears, and mucus sample of the endotracheal tube + blood smears (in one case, each; 1/153; 0.65%). Additionally, the detection of morulae on smears from pericardial fluid and pleural effusion (one case each; 1/153; 0.65%) was also reported (Table 3).

The smears were stained with different methods, the most common was Giemsa (89/153; 58.17%), followed by Diff-quick (22/153; 14.38%), Hemacolor (14/153; 9.15%), Wright–Giemsa (8/153; 5.23%), Modified Wright–Giemsa (6/153; 3.92%), Hematoxylin and eosin (5/153; 3.27%), and May–Grünwald Giemsa (2/153; 1.31%). Six blood smears (6/153; 3.92%) were stained with both Hematoxylin and eosin and Wright–Giemsa stain [34]. In one case, the type of the staining method was not mentioned (1/153; 0.65%) [43]. One buffy coat smear was analyzed using transmission electron microscopy after the blood smear from the same sample had already been analyzed by microscopy [49].

The characteristic inclusions were observed in 4% to nearly 40% of neutrophils [5,27,35,37,42,48,49], 3–4 days after tick bite [38]. Morulae were also found two weeks after their first detection in an untreated horse, which was already considered clinically recovered, but confirmation by PCR was not possible [28].

Serological methods detect antibodies against A. phagocytophilum and are of high interest in epidemiology. However, seroconversion was also demonstrated in the acute disease, in both experimentally and clinical cases, at about 12-16 days post-inoculation, including with concurrent presence of morrulae in peripheral blood [28]. Antibody titers become positive after at least 2 to 5 days after the appearance of morulae in peripheral blood and may persist for up to two years. Serological tests were conducted for 84 out of 189 cases (44.44%) by using immunofluorescence assay (IFA) (62/84; 73.81%), enzyme-linked immunosorbent assay (ELISA) (3/84; 3.57%), both IFA and Western Blot (12/84; 14.29%) and both IFA and ELISA (3/84; 3.57%). The serological method was not mentioned in four cases (4/84; 4.76%). Of the 84 samples tested, 56 were seropositive (56/84; 66.67%) (Table 3). A total of 51 samples (51/84; 60.71%) were positive for anti-A. phagocytophilum antibodies in the acute stage of the disease, 33 (33/50; 66%) were positive by IFA, 12 (12/50; 24%) by IFA and Western blot, 3 (3/50; 6%) by ELISA, and 3 (3/50; 6%) by both ELISA and IFAT. Six animals (6/84; 7.14%) were seropositive in the late stage (from 4 to 6 weeks p.i.), of which two were positive by IFA; for the remaining cases (4/6), the serological test was not provided.

Two samples (2/84; 2.38%) were positive by serological methods (ELISA and IFA) and negative by microscopic examination and PCR [33,51].

In addition, in one study, 18 of 24 analyzed samples were seropositive by IFA after 8 months [28]. Also, one horse showed ataxia and conscious proprioceptive deficits five years after the initial EGA diagnosis; microscopic examination and PCR were negative, while the serological test was positive at presentation and after 36 days [51].

The pathogen was isolated in only one case (1/189; 0.53%), using specific cellular lines and infected blood cells (confirmed by microscopic examination of buffy coat smear) preserved at −80 °C [31].

PCR detection sensitivity varies according to the assay [2]. PCR assays were reported in 122 out of 189 cases (64.55%). DNA of A. phagocytophilum was detected in 120 samples (120/122; 98.36%) by conventional PCR (120/122; 98.36%), Real Time-PCR (RT-PCR) (21/120; 17.5%), and PCR-Reverse Line Blot (PCR-RLB) (6/120; 5%).

The type of genes that were amplified were provided for 98 cases (98/120; 80.83%). The tests amplified the following genes: *16S rRNA* (81/98; 82.65%), *msp2* (14/98; 14.29%), and a portion of the multicopy pseudoankyrin gene *epank1* encoding 4 of the 11 *ankyrin* repeats, a region comprising 444 nt (1/98; 1.02%). In one case, the amplification of 2 genes was reported: *16S rRNA* and *ankyrin* protein A (*ankA*) gene (1/98; 1.02%) [40]. One sample was analyzed and reported positive by PCR assay that amplified 3 genes: *16S rRNA* gene, *ankA* gene, and citrate synthase (*gltA*) gene (1/98; 1.02%) [5]. Besides blood samples, DNA was detected from cerebrospinal fluid (1/120; 0.83%), muscle tissue (1/120; 0.83%), pericardial fluid (1/120; 0.83%), and pleural effusion (1/120; 0.83%) [41,46,50].

### 3.5. Therapeutic Management

Therapeutic management was described for 80 cases (80/189; 42.33%). Of these, 83.75% (67/80) were patients that were treated for EGA, 3.5% (4/80) were horses that received non-specific treatment before diagnosis (penicillin—3/4 and imidocarb dipropionate—1/4), and horses (9/80; 11.25%) that recovered without antibiotherapy. The type of medication, dosage, and time of administration varied among naturally *A. phagocytophilum*-infected animals. Most cases (35/67; 52.24%) were treated with oxytetracycline, but other antibiotics were also used, like tetracycline (10/67; 14.93%) and doxycycline (1/67; 1.49%). Four horses received tetracycline or oxytetracycline (4/67; 5.97%), and in another four cases, therapy was reported as a combination of antibiotics or treatment unknown (4/67; 5.97%). One horse (1/67; 1.49%) was treated with trimethoprim sulphonamide, and seven horses (7/67; 10.45%) were treated with penicillin, but in three of these cases, treatment with penicillin failed, and the therapy had to be changed. In four cases (4/67; 5.97%), therapy with oxytetracycline was followed by or switched to doxycycline, while in one case (1/67; 1.49%), minocycline was followed by and continued with oxytetracycline (Table 4).

Oxytetracycline was administered intravenously (i.v.) at doses from 3 mg/kg to 10 mg/kg for 4 to 14 days; in some cases (4/67; 5.97%), it was followed by or switched to oral doxycycline at a dose of 10 mg/kg for 5 to 14 days [46,47,50]. In one horse, oxytetracycline was administered after initiation of oral minocycline therapy, which was continued at a dose of 4.4 mg/kg for 14 days [51]. Three horses received oxytetracycline intramuscularly (i.m.) at doses of 5 mg/kg, 8.4 mg/kg, and 11.6 mg/kg (continued with 5.8 mg/kg) for 5, 10, and 7 days, respectively [30,51]. One horse was treated only with oral doxycycline on the farm, respecting the owners’ decision. Despite the fact that the treatment for this horse was discontinued after 5 days because of diarrhea, clinical signs resolved without recurrence [49].

One horse was treated with the combination trimethoprim sulphonamide and showed full recovery in 4 days [28]. Also, in four horses, treatment included drugs that were not specific for anaplasmosis until the diagnosis of EGA could be established. In three of these cases, horses were treated with penicillin before admission to the clinic and recovered spontaneously without any other treatment after 3–14 days of clinical signs [37], while one horse received a dose of imidocarb dipropionate before EGA diagnosis and recovered within five days without any other treatment [36].

Information regarding clinical improvement after therapy (p.t.) was provided for 45 out of 67 treated equids (45/67; 67.16%). Of them, 16 (35.36%) showed clinical improvement at 10–12 h p.t., 19 (42.22%) within 24–48 h, and 2 (4.44%) at or after 3 days. For seven animals (15.91) the period was not mentioned.

Data about recovery after therapy were available in 44 out of 67 cases (44/67; 65.67%); complete recovery was reported for 24 (54.55%) treated equids, at 12 h up to 8 months p.t. For the remaining 20 cases, the period was not mentioned. It can be noted that recovery for some untreated horses and for several horses treated with antibiotics other than tetracyclines was reported within the same period as in those treated with tetracycline [28].

One horse, despite i.v. administration of 7 mg/kg of oxytetracycline every 24 h, developed severe clinical signs and was euthanized on humane grounds [34]. Nine horses (9/87; 10.34%) recovered without antibiotherapy [5,28,34]; one of them developed respiratory disease subsequently [28].

## 4. Discussion

Analyzing the clinical cases resulting from the selection shows that the largest number of clinical cases of EGA was registered in Europe (127/189), and the majority of studies (20/31) were reported after 2005. A high number of EGA cases, more than 10 cases each, were from Sweden, Spain, Germany, Poland, and the Czech Republic. EGA was diagnosed in equids aged between 4 months and 30 years old, mostly males, of different pure—or crossed—breeds.

EGA develops as an acute self-limiting or subclinical disease [7,54]. Clinical signs are usually non-specific, except for limb edema and ataxia, so the fever of unknown origin should be an impulse to meticulously investigate the diagnosis for EGA [36]. In the selected articles, the most commonly observed clinical signs were fever (90.30%), limb edema (48.51%), and anorexia or inappetence (41.79%).

Experimental studies in horses reported a percentage from 0.5 to 16.0% of neutrophils with inclusion bodies [55,56]. These were first observed in neutrophils by day 12–14 post-exposure to experimentally infected ticks and from 27 days post-exposure to naturally infected ticks [55,57]. In contrast, the selected articles reported a percentage of 4% to nearly 40% of neutrophils [5,27,35,37,42,48,49], 3–4 days after tick bite [38]. After 48–72 h from the initiation of antibiotherapy in naturally infected horses, morulae are no longer present in peripheral blood. Hence, inclusions can be observed for a short period of time, which decreases the sensitivity of this diagnosis method [47]. However, morulae were still observed after 2 weeks after their first detection in a horse that did not receive any treatment, but it was considered clinically recovered [28].

In experimental studies in horses, the PCR detection period after exposure to naturally infected ticks was 3–17 days [55,57,58]. After intravenous inoculation of *A. phagocytophilum* Swedish strain, PCR was positive for a longer period, until 18–21 days [56]. In a naturally infected horse, PCR remained positive at 2 days but was negative after 28 days [51]. In the case of chronic anaplasmosis, the pathogen was detected 4 months after tick contact, so the detection period was almost equal to the one described in another experimental study, in which the pathogen persisted for at least 129 days, without any clinical or pathological abnormalities observed [41,54].

DNA of *A. phagocytophilum* can be isolated from whole blood, leukocytes, bone marrow, or fragments of the spleen [7]. In the selected publications, besides blood samples, cerebrospinal fluid, muscle tissue, pericardial fluid, and pleural effusion showed positive PCR results for *A. phagocytophilum* [41,46,50].

Anti-*A. phagocytophilum* antibodies are detected by serological methods, such as IFAT and ELISA, to determine a present or past infection. These methods are very useful in epidemiological studies, but the information obtained is not enough to establish a diagnosis in acute clinical cases [59,60]. One selected article reported seropositive horses 8 months after the acute stage of the disease [28]. Another article reported a seroconversion in a horse 2 years after it was first diagnosed with EGA, without any evidence of infection in those years [51].

*A. phagocytophilum* can be cultured from infected blood and kept for up to 18 days under refrigerated conditions [2]. In vitro culturing offers a sensitivity equivalent to that of PCR and microscopy; however, positive results are reported in approximately one week and can remain negative for more than 2 weeks [17]. Only one selected article reported the isolation of the pathogen using specific cellular lines, performed by use of infected cells preserved at −80 °C [31].

A differential diagnosis should comprise purpura hemorrhagica, liver diseases, equine infectious anemia, equine viral arteritis, encephalitis, and borreliosis [7,61]. It has also been suggested to consider EGA as a differential diagnosis for acute severe rhabdomyolysis and myalgia [46].

Co-infection with *Borrelia burgdorferi* sensu lato was also reported in six EGA cases in two studies [5,28], but all were considered most probably old infections. Of them, five horses were seropositive for *Borrelia afzelii*, but most antibody titers declined during repeated checking over a period of 12–15 months [28]), while for one Lyme borreliosis seropositive horse, discrimination between a previous and recent exposure was not pursued [5]). However, given the potential of co-infection with *B. burgdorferi* s.l., and since clinical signs of borreliosis may resemble those of EGA, this is a diagnosis to be considered. A recent serological and molecular study in Brazil (Rio de Janeiro state) reported a moderate frequency of seropositive horses for *A. phagocytophylum* (17.3%) but also simultaneous exposure to *A. phagocytophilum* and *T. equi* (18.2%). However, co-infection of *A. phagocytophilum* with *T. equi* was molecularly confirmed in only one horse, which was also the only one displaying clinical signs such as pale mucosa, cachexia, and fever [62]. In Brazil, EGA is considered an emerging disease. High seroprevalence rates have been reported in horses from the central–west region [63] and southeastern Brazil [64], of 65% (by ELISA) and 76% (by IFA test), respectively. However, characteristic inclusions of *A. phagocytophilum* were observed in 12.8% of buffy coat smears [64].

Also, serological evidence of exposure to *A. phagocytophilum* has been reported also in horses in Portugal, with a prevalence from 3% to 13% [65,66]. However, the human *A. phagocytophilum* HZ strain was found in one seropositive horse from mainland Portugal, suggesting the potential for HGA in Portugal [65].

Horses can fully recover from EGA without any treatment, but antibiotics shorten the course of the disease, and clinical signs abate after intravenous administration of oxytetracycline for 3–7 days [36,47,54]. In an experimental study, all horses diagnosed with acute EGA recovered without antibiotherapy by 21 days after inoculation of *A. phagocytophilum* strain, with negative results in PCR tests by day 22 [54]. Nine horses (10.34%) from the selected articles recovered without antibiotherapy, and some untreated horses and several horses treated with antibiotics other than tetracyclines recovered within the same period as those treated with tetracycline [5,28,34,36].

## 5. Conclusions

Clinical cases of EGA selected for meta-analysis allow for a better understanding of the clinico-pathological features. Therefore, the EGA cases were mainly characterized clinically by fever (90.30%), limb edema (48.51%), and anorexia (41.79%), while among the frequent pathological findings were thrombocytopenia (90.32%), lymphopenia (58.14%), and hyperbilirubinemia (68.97%). Diagnosis was mainly based on the detection of the pathogen by microscopy (88.44%) and/or molecular biological methods (PCR-based) (98.36%). Besides blood samples, *A. phagocytophilum* could also be detected in a cytocentrifuge preparation of the abdominal fluid (microscopic examen—M.E.), in pericardial fluid (M.E., PCR), pleural effusion (M.E., PCR), mucous sample of the endotracheal tube (M.E.), cerebrospinal fluid (PCR), and from muscle tissue (PCR). In terms of therapy and outcome, equids recovered after specific treatment with oxytetracycline, which was mostly used (52.24%), but clinical signs resolved also in one horse treated only with doxycycline. The small percentage of horses that recovered without antibiotherapy (11.25%) emphasizes the important role of monitoring EGA to initiate proper medication in order to avoid complications.

## Figures and Tables

**Table 1:** General data of the equine granulocytic anaplasmosis clinical cases (n = 189) reported in peer-reviewed published articles (1994–2022).

Country	No	Species	Sex (n)	Age (n)	Race (n)	References
**Europe**						
United Kingdom	1	horse ^Ec^	M	13 years	u.k.	[26]
Switzerland	1	horse	F	12 years	Arabian	[27]
Sweden	45	horses	M (19), F (5) u.k. (21)	1–17 years (mean: 8.2 years)	u.k.	[28]
United Kingdom	1	horse	M	23 years	u.k.	[29]
France	1	horse	u.k.	8 years	u.k.	[30]
Italy	1	horse	M	15 years	half-breed	[31]
Germany	1	horse	M	8 years	u.k.	[32]
Italy	5	horses	u.k.	2–18 years	u.k.	[33]
Netherlands	6	horses	u.k.	4 years (1), 10 years (2), 14 years (1), 15 years (2)	Dutch warmblood (5), Friesian (1)	[34]
Poland	1	horse	M	7 years	English thoroughbred	[35]
France	1	horse	u.k.	14 years	French saddle horse	[36]
Czech Republic	12	horses	M (5), F (7)	3–4 years (3), 5–11 years (9)	Warmblood (5), Thoroughbred (3), Arabian (2), Paint horse (1), Standardbred (1)	[37]
Poland	5	horses	F (3), M (2)	8 months–7 years	u.k.	[38]
Germany	14	horses	F (3), M (11)	6–20 years (12); u.k. (2)		[39]
Spain, Poland, Italy, Ukraine, Germany	30	horses	u.k.	u.k.	u.k.	[7]
Switzerland	1	horse	F	22 years	Westphalian breed	[40]
Poland	1	horse	F	12 years	u.k.	[41]
**America**						
British Columbia, Canada	1	horse	F	4 years	Thoroughbred-cross	[42]
Connecticut, New England, USA	5	horses	M (1), u.k. (4)	30 years (1); u.k. (4)	Quarterhorse-cross (1); u.k. (4)	[43]
Wisconsin, USA	1	horse	M	4 years	American Paint Horse	[44]
Grafton, Massachusetts, USA	1	horse	M	11 years	Hanoverian-cross	[45]
California, USA	1	horse	M	5 years	Quarter Horse	[46]
Virginia-Maryland, USA	1	horse	F	15 years	Thoroughbred	[47]
Nova Scotia, Canada	1	horse	M	16 years	Paint stallion	[48]
Saskatchewan, Canada	1	horse	M	30 years	Arabian-cross	[5]
Indiana, USA	6	horses	M (4), F (2)	4–22 years	Quarter (2), Paint (2), Walking (1), Arabian (1)	[49]
Massachusetts, USA	2	horse (1), donkey ^Ea^ (1)	M (1), F (1)	15 years (1), 4 months old (1)	Quarter Horse (1), Miniature donkey (1)	[50]
Virginia, USA	4	horses	F (2), M (2)	14 years (1), 21 years (1), 24 years (1), 1.5 years (1)	Przewalski’s horse (4)	[51]
**Asia**						
Pakistan	16	horses (7), mules ^Em^ (4), donkeys (5)	u.k.	u.k.	u.k.	[52]
Iraq	13	horses (13)	u.k.	u.k.	u.k.	[11]
**Africa**						
Algeria	9	horses (9/9)	u.k.	u.k.	u.k.	[53]

Legend: Ec: *Equus caballus*; Ea: *Equus asinus*; Em: *Equus mullus*; u.k.: unknown; M: male; F: female.

**Table 2:** Meta-analysis results of clinical and laboratory findings of the animals diagnosed with equine granulocytic anaplasmosis.

Parameter	Number of Studies	Number of Cases	Percentage (%)	95% CI
**Clinical signs**				
Fever	26	121/134	90.30	83.98–94.74
Limb edema	15	65/134	48.51	39.78–57.30
Anorexia	22	56/134	41.79	33.33–50.62
Depression	11	44/134	32.84	24.97–41.48
Icterus	17	30/134	22.39	15.64–30.40
Ataxia	10	24/134	17.91	11.82–25.47
Tachycardia	9	22/134	16.42	10.58–23.80
Lethargy	10	21/134	15.67	9.97–22.96
Reluctance to move	6	6/134	4.48	1.66–9.50
Abnormal movements	2	6/134	4.48	1.66–9.50
Decreased body weight	3	5/134	3.73	1.22–8.50
Debilitation	1	5/134	3.73	1.22–8.50
**Laboratory abnormality**				
Thrombocytopenia	19	84/93	90.32	82.42–95.48
* Anemia	15	30/40	75.00	58.80–87.31
** Decreased PCV/Hematocrit	10	24/34	70.59	52.52–84.91
Leukopenia	11	19/34	55.88	37.88–72.82
Lymphopenia	13	25/43	58.14	42.12–72.99
Neutropenia	4	10/24	41.67	22.10–63.36
Hyperbilirubinemia	10	20/29	68.97	49.16–84.72
Hyperfibrinogenemia	8	13/15	86.67	59.53–98.35
Hyponatremia	5	10/10	100.00	69.15–100.00

* Anemia included decreased red blood cell count, decreased hemoglobin, and/or decreased PCV/hematocrit); ** cases for which only decreased PCV/hematocrit was reported.

**Table 3:** Diagnostic methods used for diagnosis of 189 clinical cases of equine granulocytic anaplasmosis reported in peer-reviewed articles.

Diagnostic Method	Nr. of Articles (nr. of Cases in Study)	Diagnostic Method Used for EGA Clinical Cases Confirmation										References
		Morulae Identification on Microscopic Exam of Smear			Molecular PCR-Based Methods			Serology		Isolation		
		Type of Smear	Nr. Tested	Nr. Positive (%)	Nr. Tested	Nr. Positive (%)	Targeted Gene	Nr. Tested	Nr. Positive (%)	Nr. Tested	Nr. Positive (%)	
Morulae identification on microscopic exam of smear	1 (n = 13)	buffy coat	13	13 (100.00)	-	-		-	-	-	-	[11]
Morulae identification on microscopic exam of smear + PCR	13 (+2 *) (n = 88)	All types blood buffy coat pleural fluid pericardial fluid	87 70 (69 + 1) 15 (+1 **) 1 1	71 (81.60) 54 (53 + 1) 15 (+1 **) 1 1	87	87 (100.00)	epank1, 16S rRNA msp2, ankA, gltA	-	-	-	-	[5,7,29,34,35,36,38,39], [43] *, [46,47,49,50], [51] *, [52]
Morulae identification on microscopic exam of smear + Serology + PCR	9 (n = 68)	All types blood buffy coat endotracheal mucus	67 61 (59 + 2) 6 (+1 **) (1 **)	63 (94.03) 60 (58 + 2) 3 (+1 **) (1 **)	30	28 (93.3)	16S rRNA ankA	68	40 (58.82) [38 acute stage; 2 late stage (1—at 30 days; 1—at 6 wks. p.i.)]	-	-	[27,28,30,33,37,40,44,48,51]
Morulae identification on microscopic exam of smear + Serology	3 (+1 *) (n = 5)	All types blood abdominal fluid	5 5 (4 + 1) (1 **)	5 (100.00) 5 (4 + 1) (1 **)	-		-	5	5 (100.00) [3 acute stage; 2 late stage (at 4–6 weeks p.i.)]	-	-	[26], [37] *, [42,45]
Morulae identification on microscopic exam of smear + Serology + Isolation	1 (n = 1)	buffy coat	1	1 (100.00)	-		-	1	1 (100.00)—acute stage	1	1 (100.00)	[31]
Serology	1 ( +1 *) (n = 10)	-	-	-	-		-	10	10 (100.00) [9 acute stage; 1 late stage (at 3 wks p.i.)]	-	-	[43] *, [53]
PCR	3 (n = 5)	-	-	-	5	5 (100.00)	16S rRNA	-	-	-	-	[32,41,43]
TOTAL	31 (n = 189)	All types blood buffy coat cavitary fluids * endotracheal mucus	173 136 (132 + 4) 35 (+2 **) 2 (+1 **) (1 **)	153 (88.44) 119 (115 + 4) 32 (+2 **) 2 (+1 **) (1 **)	122	120 (98.36)		84	56 (66.67) [51 acute stage; 5 late stage (at 4–6 weeks p.i.)]	1	1 (100.00)	

* Articles that were counted for another diagnostic method or group of diagnostic methods; ** cases for which morulae were also detected on the blood smear (samples tested by different smear types); cases counted at the blood smear; (a + b): (a = cases for which morulae were detected only on the blood smear; b = cases for which morulae were detected on the blood smear and on other type of smear: 2 buffy coat smears, 1 endotracheal mucus smear, and 1 abdominal fluid smear).

**Table 4:** Meta-analysis results of therapy and outcome of the equine granulocytic anaplasmosis clinical cases reported in peer-reviewed published articles (1994–2022).

Variable	Number of Studies	Number of Cases	Percentage (%)	95% CI	Outcome		References
					Clinical Improvement Number of Cases)	Recovery (Number of Cases)	
Treated for EGA							
Oxytetracycline	17	35/67	52.24	39.67–64.60	- within 10–12 h (n = 16) - within 24–48 h (n = 15) - after 3 days (n = 1) - n.m. (n = 2); (1 euthanized)	- complete recovery (from 12 h to 3 months) (n = 13) - incomplete recovery (n = 1) - n.m. (n = 7); n.a. (n = 13)	[26,27,29,30,31,32,34,35,37,38,40,42,44,45,48,49,51]
Tetracycline	2	10/67	14.93	7.39–25.75	- within 24 h (n = 1) - - n.a. (n = 9)	- after 8 months (n = 8), - n.m. (n = 1)	[28,43]
Penicillin	1	7/67	10.45	4.30–20.35	- n.a. (n = 7)	- n.m. (4/7)	[28]
Tetracycline or oxytetracycline	1	4/67	5.97	1.65–14.59	- n.m. (n = 4)	- n.m. (n = 4)	[43]
Combination of antibiotics or treatment unknown	1	4/67	5.997	1.65–14.59	- n.a. (n = 4)	- n.a. (n = 4)	[28]
Oxytetracycline + doxycycline	3	4/67	5.97	1.65–14.59	- within 24 h (n = 1); - from the 3rd day (n = 1) - n.m. (n = 2)	- after 5 days (n = 1), - n.m. (n = 3)	[46,47,50]
Doxycycline	1	1/67	1.49	0.03–8.04	- within 24 h (n = 1)	- n.m. (n = 1)	[49]
Trimethoprim sulphonamide	1	1/67	1.49	0.03–8.04	- n.a. (n = 1)	- after 4 days (n = 1)	[28]
Minocycline + oxytetracycline	1	1/67	1.49	0.03–8.04	- within 24 h (n = 1)	- within 24 h (n = 1)	[51]
Non-specific treatment before diagnosis	3	11/87	12.64	6.48 -21.50	-	-	[36,37,39]
Recovered without therapy	3	9/87	10.34	4.84–18.74	-	-	[5,28,34]

Legend: n.a. = not available information regarding improvement/recovery; n.m. = period not mentioned for improvement/recovery.

## Data Availability

Not applicable.
